# Perception of Loneliness in Adolescence: Role of Maladaptive Personality Traits and Trauma-Related Symptomatology

**DOI:** 10.3390/brainsci15010086

**Published:** 2025-01-17

**Authors:** Fiorenza Giordano, Danilo Calaresi, Valeria Saladino, Valeria Verrastro

**Affiliations:** 1 Department of Human Sciences, Society and Health, University of Cassino and Southern Lazio, 03043 Cassino, Italy; 2 Department of Health Sciences, University “Magna Graecia” of Catanzaro, 88100 Catanzaro, Italy; danilo.calaresi@unicz.it (D.C.); valeria.saladino@unicz.it (V.S.); valeriaverrastro@unicz.it (V.V.)

**Keywords:** loneliness, personality, trauma, adolescence

## Abstract

Background/Objectives: Loneliness is a heterogeneous phenomenon, generally defined as an emotional experience based on the perceived distance between an individual’s actual social relationships and those he or she would like to have. Adolescence is particularly vulnerable to loneliness because of the many changes in values, feelings, and emotions that characterize it. Among the aspects that may influence this feeling of discomfort, the literature identifies maladaptive personality and a dysfunctional response to traumatic events. Our study aims to identify the possible role that maladaptive personality traits and post-traumatic stress symptomatology in the form of Post-Traumatic Stress Disorder (PTSD) and Disturbance in Self-Organization (DSO) may play in the perception of loneliness in adolescents: Methods: We identified a mediation model constructed through structural equation modeling (SEM) to test PTSD and DSO post-traumatic stress symptomatology as independent variables and maladaptive personality as a mediator in the relationship between these and perceptions of loneliness in a population of adolescents of both sexes, Italian high school students; Results: Our study identifies the significant role of PTSD and DSO symptomatology in influencing the state of loneliness, both directly and indirectly through maladaptive personality traits, which appear to exert a substantial influence on the perception of loneliness, potentially functioning as mediators in the relationship between the latter and PTSD/DSO symptomatology. Presumably, a maladaptive personality may complicate recovery from adverse and traumatic experiences by preventing the implementation of functional coping strategies and promoting dysfunctional responses. Conclusions: Our findings underscore the importance of researchers and clinicians adequately considering different personality traits and the early identification of post-traumatic stress symptomatology. A focus on adolescents’ communication and interpersonal skills and their ability to respond effectively to stressful and traumatic events may prove useful in identifying more effective strategies for preventing and managing loneliness and related distress in adolescents.

## 1. Introduction

Human beings are social creatures who flourish when they have meaningful relationships with others [1,2,3,4]. However, in today’s society, an increasing number of individuals report difficulties in meeting people, socializing, forming friendships, romantic relationships, or other supportive connections, as well as managing the relationships they already have [5,6].

In the context of the difficulties encountered when trying to meet people in the physical world, the online context seems to be the only possible tool for creating and managing relationships. However, it appears that the prevalence of loneliness is increasing globally in conjunction with a surge in social media use (SMU), as evidenced by the parallel growth in the number of individuals using social media platforms such as Facebook, Instagram, and TikTok and the sense of loneliness.

The increase in loneliness and lack of social connections may seem paradoxical in light of the constant stream of new communication technologies that offer unlimited opportunities to socialize and engage in social interactions with a greater number of interlocutors [7,8]. Certainly, online relationships can represent a new and interesting opportunity for socialization, as they are able to connect people from different countries and backgrounds, facilitating encounters, communication, and comparison, especially among adolescents and young adults, who are the main users of social media; however, many researchers have reported that these virtual relationships do not seem to be an effective antidote to the perception of loneliness, especially among younger people.

Arguably, despite the convenience and endless resources of the Internet and social media, it remains difficult to establish and maintain solid, supportive, and appreciative relationships, and as a result, an increasing number of people perceive a persistent sense of loneliness in this digital age [7,8]. Especially during adolescence, social networking sites (SNSs) seem to play a significant role in the phenomenon analyzed, with an increase in the number of young people who engage in online self-disclosure, but at the same time are enclosed and not accessible to the offline world, resulting in a greater negative impact on their levels of loneliness [7,9,10].

The concept of loneliness is inherently complex and difficult to define. Scholars have offered a variety of definitions and perspectives in describing the phenomenon [11,12,13]. The most important point of convergence among these authors is the identification of loneliness as a subjective experience, independent of the actual social and relational context in which individuals find themselves [2,6,14]. In fact, many scholars agree in defining loneliness as a form of psychological distress experienced in response to perceived deficits in one’s social relationships [7,14,15,16,17], more specifically, as the result of a perceived discrepancy in both quantity and quality [6,7,14] between desired and actual social relationships, emphasizing its difference from social isolation, considered as a concrete and physical lack of social ties [4,16,17].

The researchers also distinguish between loneliness in some respects physiological and even functional, which is a common experience that many individuals will encounter at some point in their lives, and loneliness as a maladaptive coping mechanism that is a source of suffering for individuals who experience it, affecting their current and future psychophysical well-being. They argue that the distinction depends mainly on the duration of the feeling of loneliness, that is, whether it is transient or not [4,16,18]. Indeed, loneliness is considered an intrinsic biological alarm system that plays a central role in adaptive and motivational processes [19]. It signals potential problems in an individual’s social relationships, eliciting a distress response [18,20]. This prompts individuals to invest time and energy in maintaining or repairing their social ties in order to alleviate the distress associated with loneliness [6,14,18,20]. Therefore, if the circumstances responsible for this unpleasant feeling are resolved in due time, it is unlikely to persist over time and be pervasive in all situations. Conversely, a chronic and prolonged state of loneliness may represent a maladaptive and dysfunctional coping strategy that may act as a predictor and/or fundamental maintenance factor for numerous physical and psychological complaints, resulting in significant impairment or long-term negative consequences for the health of individuals who experience it [18,20], inevitably affecting their psychological well-being and social functioning [6,14,21].

An interesting theory proposed by Hawkley and Cacioppo (2010) that highlights the profound interaction between loneliness and psychological impairment is the “loneliness loop” [2,22,23]. This theory posits that individuals who perceive themselves as socially isolated tend to perceive their environment as unsafe and potentially dangerous. Consequently, they exhibit heightened vigilance for external stimuli, which may contribute to the maintenance of their social isolation [4]. A reduction in social contact may result from the development of cognitive biases associated with the perceived unsafety of the external world and human relationships. This reinforces the individual’s social isolation, which in turn makes it increasingly challenging to establish social relationships. Consequently, the individual may attribute responsibility for his/her condition of isolation to others, which may lead to feelings of being unloved, hostility, anger, stress, low self-esteem, self-disgust, anxiety, restlessness, and despondency [7,17,24,25]. In fact, studies reveal that among individuals who complain of loneliness, it is not uncommon to engage in self-deprecating discourse, including making disparaging remarks about their abilities, comparing themselves unfavorably to others, or offering apologies for circumstances that were not their responsibility [17,20].

The literature points to a deep interconnection between the perception of loneliness and a range of psychological disorders, including depression and anxiety, self-harm, suicidal ideation [5,16,18,25], disordered eating [11,12], and substance addictions and dysfunctional behaviors [26,27], as well as post-traumatic stress disorder (PTSD) and complex PTSD symptomatology [22,23,28,29].

The latest version of the World Health Organization’s International Classification of Diseases 11th Revision (ICD-11) [30,31], published in 2018 and effective from January 2022, includes a new definition of post-traumatic stress disorder (PTSD) consisting of six symptoms and a new diagnosis of complex post-traumatic stress disorder (CPTSD) that covers twelve symptoms (six PTSD symptoms and six “disturbance in self-organization” [DSO] symptoms), each organized in three clusters of symptoms. The PTSD clusters encompass the re-experiencing of the traumatic event in the present, avoidance of reminders of the trauma, and a sense of current threat. CPTSD is characterized by the presence of the three PTSD clusters and three additional symptom clusters that reflect the disturbance in self-organization (DSO): affective dysregulation, negative self-concept, and disturbances in relationships. In order to meet the criteria for a diagnosis, both PTSD and CPTSD must be accompanied by traumatic exposure and a notable decline in functioning [13].

As evidenced in the literature, there is a potential correlation between loneliness and an increased likelihood of manifesting symptoms of Post-Traumatic Stress Disorder (PTSD) and Complex Post-Traumatic Stress Disorder (CPTSD) through various proposed pathways, among which, a considerable number of authors have identified a kind of cyclical relationship between the distress caused by loneliness and that caused by stressful and traumatic experiences [15,20,28,29]. For instance, research has indicated that both conditions share a number of negative cognitive biases, particularly a state of hypervigilance resulting from the perception of continuous threats, regardless of the presence of risks and dangers in the environment [20]. Other common symptoms reported by individuals in both conditions include re-experiencing/intrusion symptoms, avoidance symptoms, negative evaluations of the world, experience of self-disgust, poor sleep quality, and feelings of alienation, detachment, and estrangement from others [17,32].

Some researchers have observed that loneliness hinders the process of coping with and overcoming traumatic experiences, particularly by prolonging and complicating the natural recovery process after bereavement [7,20,28,29]. The relationship between loneliness and PTSD symptomatology may be understood in two ways. It is evident that a state of loneliness increases the probability of developing PTSD and the duration of its symptoms. In fact, loneliness can lead to social withdrawal [2,4], which can be used as a coping strategy in response to PTSD symptoms, which actually ends up perpetuating PTSD symptoms [32,33].

However, the reverse process may also occur, whereby PTSD symptoms, particularly social withdrawal and relationship difficulties, can lead to behaviors that result in social isolation and intensify feelings of loneliness [17,33].

Based on the results of the above studies, it was decided to conduct further investigation into the potential impact of symptomatology associated with post-traumatic stress disorder (PTSD) and disturbance in self-organization (DSO) on the perception of loneliness. In light of the existing literature, it can be posited that one of the most interesting factors associated with trauma response symptomatology and the perception of loneliness is the influence of personality traits, particularly maladaptive ones.

In fact, in accordance with Osler’s (1899) assertion that “It is more important to know the person with the illness than the illness the person has”, it may be beneficial to direct attention not only to the symptoms of psychological disorders but also to the underlying personality-related propensity for psychopathology [34]. This approach could enhance both prevention and care interventions by focusing on the specific characteristics of the individual rather than solely on the psychopathology features and symptomatology.

Some scholars have demonstrated that loneliness and personality are closely interrelated, with an association between loneliness and each of the Big Five personality traits [15,35]. The findings indicate a significant association between loneliness and lower perceived conscientiousness, extraversion, and agreeableness, as well as higher neuroticism [15,35]. Conversely, it appears that there is no significant association between loneliness and openness to experience [35], even if the assumption that loneliness is closely related to personality characteristics does not preclude the possibility that there are some individuals who are lonely but nevertheless able to interact effectively in a number of contexts and situations.

In accordance with Costa and McCrae’s (1980) five-factor personality model (FFM), the most consistent relationships with loneliness and well-being were identified for neuroticism and extroversion [35,36]. The first was identified as the strongest correlate for reduced well-being and enhanced loneliness. In fact, neuroticism is defined as a personality trait characterized by a tendency to experience negative emotions and a heightened level of distress in response to changing life circumstances. Those who are emotionally unstable (i.e., individuals high on neuroticism) experience fear, depression, and guilt more frequently than those who are emotionally stable and are also more sensitive to cues of social rejection as well as more susceptible to stress and changes in life situations. Consequently, they tend to have more dysfunctional interpersonal relationships and are less satisfied with their relationships. Extraversion, in turn, is associated with engaging in and deriving pleasure from social interactions, participating in social activities, and having a larger number of social connections. Individuals with a high level of extroversion tend to receive more social support from others, suggesting that they can count on more social support when facing stressful or adverse events. Furthermore, individuals high in extraversion demonstrate a greater capacity to experience a positive effect and to maintain it over time, particularly in the context of emotionally ambivalent situations. Extroverts, therefore, typically show a reduced sense of loneliness and greater well-being than introverts, for whom maladaptive cognitive strategies, such as rumination or catastrophizing, were found to be associated with high levels of loneliness [35,36].

In 2013, the American Psychiatric Association introduced an alternative model for personality disorders in the DSM-5 (Diagnostic and Statistical Manual of Mental Disorders, 5th edition) which includes five maladaptive personality traits [37,38]. These domains are regarded as extreme variants of the five-factor model. Negative affectivity, which may be conceived of as the opposite of emotional stability, is associated with a range of emotional states and behaviors, including anxiety, depression, and anger. The trait of detachment (which is the opposite of extraversion) is expressed by the avoidance of social or emotional situations through social withdrawal and the restriction of affectivity. Those with elevated scores in antagonism (the polar opposite of agreeableness) tend to engage in behaviors that precipitate interpersonal discord. Individuals with high scores in disinhibition (the opposite of conscientiousness) tend to prioritize immediate gratification and engage in impulsive behavior. Finally, psychoticism (the inverse of openness) is associated with inappropriate or unconventional conduct, as well as hallucinatory phenomena and peculiar inner experiences [39,40].

Maladaptive personality traits have been demonstrated to frequently co-occur with interpersonal difficulties. In fact, the literature suggests that individuals with maladaptive personalities often encounter significant challenges in their interpersonal relationships, which can lead to increased anxiety, feelings of inadequacy, and social isolation [20,41].

Maladaptive personality traits are associated with an increased risk of psychological distress due to their tendency to be positively correlated with avoidant/maladaptive behaviors and negatively correlated with acceptance and positive reframing, which are forms of coping [32,42].

For instance, among the elderly, personality traits of psychoticism and antagonism emerge as particularly potent predictors of relationship difficulties [43]. Additionally, certain personality traits among adolescents and young adults may impede their ability to form connections with others, potentially leading to feelings of loneliness. These traits include shyness, introversion, unsociability, and neuroticism [7].

Regardless of age, individuals with high levels of abnormal personality dimensions often show a tendency to avoid social situations and activities and may prefer solitude even in the presence of others [44].

Some scholars identify an association between maladaptive personality traits and the presence of internalizing distress symptoms in adults who have experienced childhood trauma [34,45]. Others point to a correlation between maladaptive personality traits and substance abuse and/or dysfunctional behaviors, particularly Internet-related disorders, gaming disorders, and binge-watching [45,46]. These disorders seem to be related to high negative affectivity and disinhibition. The former is characterized by a preponderance of negative affective states, including anxiety, sadness, anger, and other dysphoric moods. The second is suggestive of a propensity for immediate gratification and impulsive behavior. Both aspects are key features of addictive behaviors and serve as primary maintenance factors [47].

Other psychological discomforts associated with maladaptive personality traits include problematic eating behaviors. For example, the dimension of impulsivity has been found to interact with personality traits in predicting orthorexia, as suggested by Awad, E., et al. (2022), who emphasize the role that female gender, maladaptive personality traits, and impulsivity play in the development of orthorexic eating behaviors [48].

In a recent study, Reinhard, M.A., et al. (2022) found that patients with personality disorders (PDs) frequently report feelings of loneliness and disconnection [49]. Additionally, the study revealed that, compared to individuals with other mental health diagnoses, patients with PDs were the most affected by loneliness.

Personality disorders (PDs) are defined by enduring patterns of inner experience and behavior that markedly deviate from the individual’s cultural norms and expectations, appearing inflexible, stable, distressing, and negatively affecting cognition, affectivity, interpersonal behavior, and/or impulse control, resulting in impaired personal and interpersonal functioning. As a result, patients with PDs have difficulty establishing and maintaining functional social relationships [49].

Comparison between personality traits and feelings of loneliness is possible thanks to studies such as the one conducted by Mund, M., et al. (2020), who showed that over the life span, the ranking order of loneliness is as stable as that of personality traits [14] and the one designed by Buecker, S., et al. (2020), who highlighted the importance of stable personality factors in explaining individual differences in loneliness [35].

In our study, we aim to explore the adolescent population to understand how much the condition of loneliness is perceived and suffered by younger people, in order to identify the factors that seem to accompany and/or promote it and whether and how the perception of loneliness may interact with other aspects of adolescents’ lives, with particular attention to personality traits that may disfavor socialization and communication processes and the impact that problematic or traumatic experiences through the development of PTSD or DSO symptomatology may have on the condition of adolescent loneliness. Although all age groups may experience the unpleasant feeling of loneliness, the literature suggests that this phenomenon may be particularly pronounced in the elderly and adolescents [14]. Often the elderly, after retirement, may suffer from the absence of their socio-working environment and colleagues, or they may lose their landmarks and habits due to the death of friends or partners, or the distance from their children [22,23,50]. Similarly, adolescence is a phase of life that involves changes, particularly in the dynamics of interpersonal relationships [21,50,51]. These changes are significant both in terms of meaning and relevance, as adolescents move away from their parents, who are no longer considered a point of reference and often become the object of conflicting relationships [4,25]. In addition, the transition to a new school with new teachers and classmates, the loss of childhood friends, and the experience of first romantic relationships can all contribute to intricate interpersonal relationships and often generate experiences of loneliness [4,23,51]. During adolescence, the condition of loneliness is associated with a number of social and psychological discomforts including an increased risk of alcohol and substance addiction [27], a greater likelihood of developing depressive symptoms in young adulthood, and an elevated probability of experiencing disability and lower income in midlife [4,21].

The literature indicates that a constellation of adversities, including stressful and traumatic experiences, may be predictive of loneliness in adolescents [7,9,25].

These include psychological maltreatment and victimization, social ostracism, negative affective experiences including bullying from peers and siblings, arguments with parents, and family conflict [1,7]. Additionally, loneliness in adolescence has been linked to parental conflictual relationships and divorce, having an ill family member, poor student-teacher relationships, and problematic use of social media [1,7,25]. A common phenomenon that strongly links bullying and the problematic use of social media consists of cyberbullying, in which adolescents may take on the dual role of victim and perpetrator, thus demonstrating less ability to cope with stress and a greater degree of loneliness [7,10].

Adolescents who experience loneliness often perceive themselves and their role in social relationships in a negative manner. This perception may contribute to a state of mind that hinders social contact. Cacioppo’s evolutionary theory posits that loneliness in adolescence is a consequence of heightened social sensitivity in response to the social challenges of adolescence, which among adolescents leads to a self-focused orientation that impairs their capacity to socially reconnect [4]. More than in adulthood, loneliness may be a functional and adaptive mechanism for adolescents to establish their place in the world. However, as with adults, when this condition persists, it can impair social and interpersonal skills, increase hypervigilance and social avoidance, and reduce trust in others. This is done to protect themselves from fear or even the expectation of being rejected, isolated, or humiliated [4]. This attitude can greatly disrupt interpersonal relationships, intensifying the painful perception of loneliness and social isolation. In particular, the difficulty in trusting others and, in some cases, the inability to trust anyone, seems to be a significant factor contributing to the perpetuation of a vicious cycle of loneliness that can last for months or even years [15,25,51].

Based on the literature reviewed, we hypothesized that the presence of PTSD and/or DSO symptomatology may have an impact on the perception of loneliness in adolescence and that this interaction may be reinforced where maladaptive personality traits are observed. To test these hypotheses, we decided to conduct a mediation analysis using structural equation modeling (SEM), identifying PTSD and DSO symptomatology as the predictors, maladaptive personality traits as the mediator, and perceived loneliness as the outcome.

## 2. Materials and Methods

### 2.1. Study Design

In the present study, we performed a preliminary correlation analysis among all the variables under our study using Pearson’s product moment correlation coefficients and then tested a mediation model calculated through Structural Equation Modeling in order to examine whether maladaptive personality was a potential mediator in the relationship between the independent variables identified in post-traumatic stress symptomatology, in the form of PTSD and DSO, and the dependent variable represented by the perception of loneliness.

### 2.2. Setting

Recruitment was carried out through an email invitation to school principals to participate in the research project. The e-mail described the characteristics and objectives of the study, the recruitment methods, the questionnaire administration methods, and the rules for informed consent. The latter was given to the students in paper form by the teachers with a request to return it signed by their parents or guardians. Students who did not return the signed consent form were automatically excluded from the study. As the questionnaires were to be administered online, it was necessary for each student to have an electronic tool, a smartphone, tablet, or PC.

Students who took part in the research were asked to fill out a self-report questionnaire, which was administered online and took about 15 min to complete. The form contained a description of the characteristics and purpose of the research, as well as the informed consent form that previously their relatives/guardians had to sign in order to view the questionnaires.

In accordance with the international guidelines of the Declaration of Helsinki 1964, last revision in 2000, and the ethical code of the Italian Association of Psychology (AIP), participation in the questionnaire administration phase was subject to the signature of an informed consent form by each participant and their parents/guardians. Participation was voluntary and no prizes or compensation were offered. Privacy was ensured at all stages of the study.

The study was approved by the Institutional Review Board of the Institute for the Study of Psychotherapies, School of Specialization in Brief Psychotherapies with a Strategic Approach (reference number: ISP-IRB-2023-5, 9 January 2023).

### 2.3. Participants

The study targets a population of adolescents aged 14–17 years of both sexes, recruited by the snowball sampling method through the involvement of high schools in the Italian territory that responded to the e-mail invitation to collaborate in the research by expressing their interest and willingness to participate. The only inclusion criteria were an age between 14 and 17 years and adequate knowledge of the Italian language.

### 2.4. Variables

A mediation analysis was drawn through structural equation modeling (SEM). A structure with PTSD and DOS, MP (Maladaptive personality), and LP (Loneliness Perception) as latent variables was used to examine a model in which PTSD and DOS are considered as predictor variables, MP as mediator, and LP as outcome.

### 2.5. Measurement

To measure perceived loneliness, we used the UCLA Loneliness Scale (Version 3) [24] a 20-item measure that assesses how often a person feels disconnected from others. The questionnaire contains only a few items that could be seen as asking directly about feelings of loneliness (e.g., “How often do you feel alone?”, “How often do you feel isolated from others?”), and instead focuses primarily on participants’ ratings of various qualitative features of their social networks (e.g., “How often do you feel part of a group of friends?”, “How often do you feel that there are people who really understand you?”). The scale is rated on a four-point Likert scale (from 1 = never to 4 = always), and the total score is given by the sum of all responses ranging from 20 to 80. Results indicated that the measure was highly reliable, both in terms of internal consistency (coefficient alpha ranging from 0.89 to 0.94) and test-retest reliability over a 1-year period (r = 0.73) [24,52,53].

The assessment of post-traumatic stress disorder (PTSD) and disturbance in self-organization (DSO) symptoms was conducted using the International Trauma Questionnaire (ITQ), which is the only validated self-report measure for the assessment of ICD-11 PTSD and Complex PTSD/DSO symptomatology [54,55,56]. The ITQ initially requests that the participant identify the most distressing traumatic event and the time elapsed since its occurrence. The International Trauma Questionnaire (ITQ) comprises six items for each of the PTSD symptoms, which are grouped into three clusters: re-experiencing (e.g., experiencing the traumatic event as if it were happening again in the present), avoidance (of both internal and external reminders), and sense of current threat (e.g., hypervigilance, hyperarousal). Each cluster is represented by two items. Participants are asked to indicate the extent to which they have been bothered by each symptom over the past month. Furthermore, participants indicated the extent to which these symptoms have impeded their ability to function in daily life over the past month, as measured by an additional three items. The assessment of Complex PTSD comprises the three PTSD clusters and three additional symptom clusters that reflect DSO, namely affective dysregulation, negative self-concept, and disturbances in relationships. The questions pertain to the respondents’ typical emotional states, self-perception, and interpersonal dynamics. Additionally, three items are employed to assess the extent to which these symptoms impede functional capacity in the previous month. All ITQ items are based on a five-point Likert scale, ranging from 0 (not at all) to 4 (extremely).

The diagnosis of post-traumatic stress disorder (PTSD) is based on the presence of trauma exposure and the endorsement of at least one symptom from each of the three symptom clusters, as well as the endorsement of at least one indicator of functional impairment. The endorsement, which is defined as a score of 2 or above on the Likert scale, indicates the presence of a particular symptom. A diagnosis of Complex PTSD necessitates the endorsement of one of two symptoms from each of the three PTSD symptom clusters and one of two symptoms from each of the three Disturbances in Self-Organization (DSO) clusters. In order to meet the criteria for a diagnosis of PTSD or Complex PTSD, it is necessary to identify at least one indicator of functional impairment related to the symptoms of PTSD and at least one indicator of functional impairment related to the symptoms of DSO. The reliability of the measure was α = 0.88 for both the PTSD and DSO subscales.

Maladaptive personality traits were evaluated using the Personality Inventory for DSM-5 (PID-5; Krueger et al., 2011) a provisional assessment instrument developed to correspond with a maladaptive personality trait model for DSM-5 [57].

The Personality Inventory for DSM-5—Brief Form (PID-5-BF) is a 25-item self-rated personality trait assessment scale. The instrument identifies five domains of personality traits, including negative affect, detachment, antagonism, disinhibition, and psychoticism. Each domain is composed of five items. The PID-5-BF employs a self-report methodology, wherein respondents are asked to rate the extent to which each item describes their own characteristics. Each item on the measure is rated on a four-point scale, with 0 indicating a response that is “very false” or “often false” and 3 indicating a response that is “very true” or “often true”. The overall measure has a range of scores from 0 to 75, with higher scores indicating greater overall personality dysfunction. Each domain of the trait scale ranges in score from 0 to 15, with higher scores indicating greater dysfunction in the specific domain of the personality trait. The mean total score is calculated by dividing the raw overall score by the total number of items in the measure (i.e., 25).

The reliability and construct validity of the Personality Inventory for DSM-5 Brief Form (PID-5-BF) were also assessed by Fossati and colleagues on a sample of high school students in 2017, demonstrating Cronbach’s alpha values for the PID-5-BF total score of 0.83 and adequate temporal stability after a two-month test-retest interval, as indicated by intraclass r values ranging from 0.78 (Negative Affectivity) to 0.97 (Detachment) [58].

All of the variables analyzed are quantitative, as evidenced by the exclusive use of self-report survey instruments, and all of the questions that make up the above questionnaire were defined as mandatory to ensure no missing data.

Each of these instruments has been validated in Italy and has been previously administered and analyzed in samples of adults and adolescents, demonstrating good levels of reliability and validity [52,56,58].

These results were confirmed by the results of our model, as shown in Table 1.

### 2.6. The Statistical Methods

Preliminary statistical analyses were conducted using IBM SPSS (Statistic Package for the Social Sciences) version 29. These included a descriptive analysis of the main characteristics of the sample and a correlational analysis of the identified variables using Pearson’s product moment correlation coefficients with a bias-corrected and accelerated 95% confidence interval (CI) (BCa). The internal reliability of each selected instrument was tested through the use of Cronbach’s α and the normal distribution through skewness and kurtosis values [59]. The results are presented in Table 1.

R Studio version 4.3.2 with the lavaan package for R was utilized to assess the factorial validity of the employed measures, regression analysis, and the structural equation modeling (SEM) for the identified mediation model.

In the SEM analysis, we used the parcellation approach; this was done for several reasons. First, this method reduces model complexity, improves normality (plots tend to have more normal distributions than individual items), increases long-term sustainability, and ensures more stable parameter estimates. In addition, the plots not only meet normality assumptions more easily but are also less susceptible to the influence of method effects. In the present study, three plots were constructed for each observed and latent variable.

**Figure 1:**
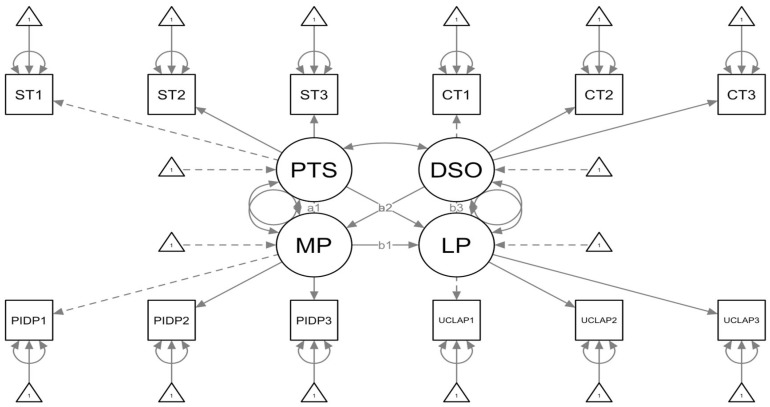
This figure outlines the SEM Path of the selected model, determined through the parceling approach. Specifically, in the plot, circles represent latent variables, rectangles represent observed variables, and triangles represent measurement errors. PTS = Post-traumatic Stress Disorder with ST1, ST2, ST3 representing its three constituent parcels; DSO = Disturbance in Self-Organization with CT1, CT2, CT3 representing its three constituent parcels; MP = Maladaptive Personality Traits with PIDP1, PIDP2, PIDP3 representing its three constituent parcels; LP = Perception of Loneliness with UCLAP1, UCLAP2, UCLAP3 representing its three constituent parcels. a1 is the direct path between Post Traumatic Stress Disorder (PTS) and Maladaptive Personality (MP); b1 is the direct path between Maladaptive Personality (MP) and Perception of Loneliness (LP); a2 represents the direct path between Post Traumatic Stress Disorder (PTS) and Perception of Loneliness (LP); b2 indicates the path between Disturbance in self-organization (DSO) and Maladaptive Personality (MP); b3 represents the direct path between Disturbance in self-organization (DSO) and Perception of Loneliness (LP). For more details, see Table 3.

The resulting model is shown in Figure 1.

## 3. Results

### 3.1. Participants: Demographic Characteristics

The study population, selected using convenience sampling, consisted of 901 adolescents aged 14–17 years (minimum age 14 years, maximum age 17 years, mean age 15.69 years, standard deviation 1.085 years) of both sexes, of whom 493 were female (54.7 percent). Regarding the sample’s gender/sex identification, participants were asked to identify their male/female biological sex and, in a follow-up question, were given the opportunity to select a third non-binary option to identify their gender identity. In the latter category, 1.6 percent of participants indicated that they identified as a non-binary gender. Both questions were mandatory.

In terms of other demographic characteristics, 95.9% of participants reported that their sociocultural background was Italian. Most participants (79.5%) live with married or cohabiting parents. Furthermore, 47.1% of participants’ mothers or equivalent and 46.8% of participants’ fathers or equivalent had a high school diploma.

### 3.2. Descriptive Data

The decision to focus our analysis on the adolescent age group is dictated by the goal of examining a phase of individual development that more than others is recognized in the literature as particularly vulnerable to the perception of loneliness. This is due to the significant transitions that occur during this period of life involving physical appearance, psychological factors, personal identity, educational environments or access to the world of work, interpersonal relationships both in terms of type and intensity with parents, peers, teachers, and early love experiences. Regarding the analysis of the demographic characteristics of the selected sample, it is important to note that in the statistical analyses, we considered only the participants’ self-reported biological sex, while the other demographic characteristics were not subject to statistical analysis, except for frequencies and descriptive analyses. Among the characteristics of the selected sample, one of the most important and sensitive aspects that was not included in the analyses was gender identity. In defining and dividing the sample by sex, we considered only the binary male/female sex. The decision not to include the variable of gender identity stems from the knowledge that this topic deserves care and attention that our study probably could not have ensured since this variable was not included in the focus of the current research design. Since gender identity is a very complex and broad concept, in order to study it properly it would have been necessary to define and specify it further, first by trying to better clarify the meaning of the related question, second by adding many more options from which individuals could choose, and finally by allowing participants to choose not to answer this question at all.

### 3.3. Outcome Data

All data were analyzed using IBM SPSS (Statistic Package for the Social Sciences) version 29 and RStudio version 4.3.2 with the lavaan package.

All variables showed statistically significant mutual correlation with a *p*-value < 0.001, as shown in Table 1.

In addition, the internal reliability and normal distribution of the data for each instrument used were verified through the use of Cronbach’s α, Skewness, and Kurtosis.

In addition, the model was evaluated on the basis of multiple indices of fit as suggested by Bentler and Bonett (1980) [60].

All fit indices were found to be adequate, with Comparative Fit Index (CFI) and Tucker–Lewis Index (TLI) values above 0.95 and 0.94, respectively [60].

In addition, the SRMR (Standardized Root Mean Square Residuals) and RMSEA (Root Mean Square Error of Approximation) indices were also satisfactory, as they were below the maximum thresholds recommended by Schermelleh-Engel et al. (2003) [61], with values of 0.03 and 0.08, respectively.

All fit indices values, including X^2^ values and degrees of freedom, are presented in Table 2.

### 3.4. Main Results

The findings of Pearson’s correlation coefficient analysis suggest a high degree of significance in the correlation between all the variables examined, with particularly robust correlations observed between the personality trait of psychoticism and loneliness perception (r = 0.520, *p* < 0.001) and psychoticism with PTSD (r = 0.531, *p* < 0.001), and DOS (r = 0.567, *p* < 0.001). Additionally, significant correlations were observed between Negative Affectivity and PTSD and DOS, as well as between Detachment and Loneliness Perception (r = 0.540, *p* < 0.001) and Detachment and DSO (r = 0.511, *p* < 0.001). The correlations of antagonism and disinhibition with all of the analyzed variables, i.e., loneliness perception, PTSD, and DSO, were less robust than those observed for the other variables, but still statistically significant. Furthermore, a marginally stronger correlation was noted between the total score of maladaptive personality traits and loneliness perception with DSO symptoms (r = 0.631, *p* < 0.001), (r = 0.671, *p* < 0.001) than the correlation between the same and PTSD symptoms (r = 0.571, *p* < 0.001), (r = 0.469, *p* < 0.001).

In constructing the mediation model, with regard to the independent variables consisting of PTSD and DSO symptomatology, we used the International Trauma Questionnaire divided into the six items constituting PTSD symptomatology, plus the three items related to the extent to which these symptoms interfered with the individual’s ability to function in daily life in the past month, and the six items reflecting DSO symptomatology, with the additional three items assessing the extent to which these symptoms interfered with the individual’s ability to function in the past month. Accordingly, we considered two different indirect variables: PTSD symptomatology and DSO symptomatology. We chose not to include Complex Post-Traumatic Stress Disorder (CPTSD), and thus not to integrate PTSD scores with DSO scores, as assessing this distress would require clinical instruments and interviews that are beyond the scope of our study, which does not pursue diagnostic purposes.

The construct thus determined proved to be sufficiently stable and robust with satisfactory fit indices, especially for the DSO scale.

As for the mediator, which in our model is represented by maladaptive personality, we used the Personality Inventory for DSM-5 (PID-5) questionnaire, which was included in the form of a total score rather than in the form of five different scores, one for each of the five domains that make up the questionnaire. The choice to use only the total score of the questionnaire arose from the intention to observe how maladaptive personality as a whole can act directly on the dependent variable consisting of the perception of loneliness and as a mediator in the indirect relationship between the latter and the predictor variables. Only in Pearson’s correlation analysis did we decide to examine maladaptive personality both as a total score and in the form of the five different scores specific to each personality trait, to monitor which of them showed greater interaction with the predictive and dependent variables.

Finally, with regard to the dependent variable represented by the perception of loneliness, we used the UCLA Loneliness Scale (version 3) in its total score, given by the sum of the responses of all 20 items that make up the questionnaire.

Since this construct showed good fit indices and the resulting model appeared robust and stable, it was decided to use it as the final model for the mediation analysis.

The results that the model thus identified reported in the structural equation modeling analysis indicate that the direct effects of the predictor variables, namely PTSD and DSO symptomatology, on the perception of loneliness are significant and seem to confirm the results of previous studies and the initial hypotheses proposed in this research.

Moreover, the indirect effect of PTSD and DSO on the perception of loneliness through maladaptive personality traits was shown to be statistically significant, thus confirming our initial hypotheses.

**Figure 2:**
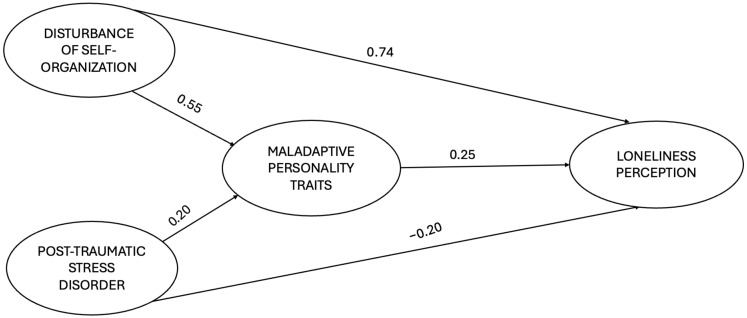
The figure portrays the mediation model that was utilized. For purposes of clarity, only the direct paths are reported; parcels are not reported.

The results are presented in Figure 2 and Table 3.

### 3.5. Other Analyses

In order to investigate the influence of sex on the selected variables, it was decided to conduct an additional mediation analysis by introducing biological sex as a predictive control variable, to see if this could affect the mediation model both in terms of model fit indices, i.e., influence on model stability and robustness, and in terms of altering the direct paths previously found for the entire undifferentiated sample with respect to the sex variable. The analysis showed that the sex variable had no effect on the stability and goodness of fit indices of the model, as indicated by CFI = 0.96, TLI = 0.94, RMSEA = 0.80, and SRMR = 0.50, and did not appear to have a significant effect on the direct paths of the variables analyzed, with values of (β = 0.00, *p* = 0.992) and (β = −0.04, *p* = 0.061) for maladaptive personality and perceived loneliness, respectively. For the reasons discussed above, only the biological sex variable was included in this analysis. Therefore, 1.6% of participants who answered the gender identity question by selecting the third nonbinary option were included in this analysis based on their responses to the biological sex question in the male/female binary code.

## 4. Discussion

The research results seem to confirm our initial hypotheses, showing that the presence of symptomatology related to PTSD and DSO disorders can directly and indirectly influence, through maladaptive personality traits, the perception of loneliness among adolescents aged 14–17 years.

The fit indices of the model are all satisfactory. The direct effect of both PTSD and DSO symptoms on loneliness, as well as the direct effect of maladaptive personality traits on loneliness perception, are significant (*p* < 0.001), thereby supporting the findings of previous literature and the hypotheses on which our research design was based. The indirect relationship between both PTSD and DSO symptomatology and loneliness, which is mediated by maladaptive personality traits, is also significant, thereby confirming the initial hypotheses.

As a social species, humans are dependent on a secure and supportive social environment to ensure their survival and well-being [3,15,20]. The experience of social isolation or loneliness has been demonstrated to increase an individual’s vigilance in the presence of potential threats, thereby heightening feelings of vulnerability [4,15,20].

The experience of inadequate social ties and the perceived lack or absence of close and supportive relationships that can be counted on, accompanied by the need to belong and the desire to feel part of a social, peer, or family group from which to gain recognition and social support, can exacerbate the consequences related to traumatic and stressful experiences. Individuals who have experienced traumatic or stressful events often report a sense of estrangement from the external world and perceive human relationships as remote or unattainable. For instance, experiences of derealization may be accompanied by a longing for close attachments and a sense of belonging within a larger network, which are also hallmarks of loneliness.

Our results seem to confirm the assumptions in the literature and the initial hypothesis from which our research started, namely, that PTSD and DSO symptomatology can play a significant role in generating, maintaining, and reinforcing the condition of loneliness, contributing to transforming a transient experience common to many human beings especially at specific stages of their lives and in some respects functional to their psychological and social well-being into a vicious cycle of suffering that threatens the individual’s physical and mental health by becoming pervasive in every aspect of their lives.

An other important hypothesis underlying our study, which seems to be confirmed by the results obtained, is the supposition that maladaptive personality traits may play a significant role in directly influencing the perception of loneliness and in acting as mediators in the relationship between the latter and PTSD/DSO symptomatology, probably in that they may complicate recovery from adverse and traumatic events experienced, hindering the implementation of functional coping strategies and increasing dysfunctional responses. These responses may consist of attitudes and behaviors characterized by isolation and closure or a prevalence of impulsivity that may evolve into substance abuse, eating disorders, behavioral addictions such as gambling, dysfunctional behaviors such as binge-watching, problematic social media use, Internet gaming, etc. to reduce unpleasant feeling [7,45,46]. Negative affectivity deteriorates feelings of trust in others, complicating the building and maintenance of satisfying interpersonal relationships [51]. In addition, individuals in whom this personality trait is prevalent tend to experience great anxiety, anger, or feel down and hopeless, all of which undermine their ability to deal with adverse events and to reacquire self-confidence, trust in others, and self-efficacy. The personality trait of detachment is naturally related to social isolation. Individuals with a high level of this personality trait tend to exhibit a strong tendency to avoid social contact and interpersonal relationships. The psychotic personality trait does not help to overcome traumatic experiences and reduce PTSD- and DSO-related symptomatology; in fact, strange inner experiences such as hallucinatory phenomena and/or paranoid aspects, i.e., the belief that one is the object of ridicule or persecution, make social interactions almost impossible. Antagonism is considered a dysfunctional personality trait, as individuals who predominantly exhibit this personality trait tend to interpret and experience interpersonal relationships as highly conflictual, overly competitive, and consequently, as a source of problems and discomfort rather than a source of support. Disinhibition may represent a personality trait that may drive the individual to implement dysfunctional coping strategies stemming from impulsivity and the compelling need for gratification that may be embodied in dysfunctional behaviors such as the abuse of the Internet, social media, sports and physical activity, etc., or that may evolve into mental disorders such as substance addiction, eating disorders, etc., which serve as self-medication to reduce intolerable moods and to achieve a sense of gratification and well-being as soon as possible [62,63].

Scholars point out that some of the characteristics and symptoms of PTSD, particularly those related to avoidance of interpersonal relationships and DSO symptoms associated with difficulty maintaining close relationships, can lead to increased feelings of loneliness [17,18,23]. Based on these assumptions, all the dysfunctional personality traits seem to share some important characteristics with the symptomatology associated with PTSD and DSO disorders, from isolation and sociability to alcohol and other substance abuse, from emotional overreactions, such as uncontrolled anger and/or extremely low mood, to dissociative experiences such as depersonalization and/or derealization, to the tendency to regard people with whom one is in contact as unreliable, antagonistic, enemies, etc.

A significant percentage of individuals are likely to experience transient and intermittent episodes of loneliness at some point in their lives. If the underlying causes are addressed early, these episodes are unlikely to persist over time and become pervasive in all situations [15,16,18,20,25]; The literature points out that loneliness can prolong over time until it becomes chronic [20,51] in individuals who tend to be hypersensitive to the opinions, judgments, and expectations of others and suffer greatly from any feedback perceived as non-positive. Fearing possible criticism, rejection, or disapproval, these individuals tend to experience interpersonal relationships and social interactions in a constant state of alertness, becoming hypervigilant to social threats and paying attention to potential negative social feedback to avoid future distress [20].

Loneliness creates negative expectations about social encounters and the intentions of others, leading to guarded behaviors and social difficulties [15,41]. The presence of guarding behaviors reinforces these maladaptive beliefs, further exacerbating the disconnect between individuals and their social context. Social withdrawal may also occur, in which the individual observes others from afar and worries about how to interact with others, instead of actually experiencing social relationships [4,25]. When an individual enacts an avoidant approach to socialization, it can elicit negative reactions from others, which in turn increases withdrawal from social interactions, creating a vicious cycle of loneliness [20,51].

The transition from childhood to adolescence is marked by a major shift in emotional support as children begin to seek autonomy from parents who previously provided such emotional support [4,7,25]. During this period, adolescents also feel the need for a greater level of intimacy in their friendships as they begin to explore their identity and undergo changes in values and beliefs. They also begin to acquire new perspectives through which to observe and evaluate themselves and others [15,20].

In other words, adolescence is one of the developmental periods at greatest risk of loneliness [7,50,51]. During this period, adolescents manage more complex relationships as they move away from the protection of the family [15]. The desire for autonomy and self-determination, coupled with the anxiety of taking on greater responsibilities and commitments, cognitive maturation, and physical changes, are all inherent features of this stage of life that could facilitate the perception of loneliness [4,20,25,51].

Some studies have found that people who perceive higher levels of loneliness are described by those closest to them as having low self-esteem, insufficient self-awareness, and shyness, with a tendency to use self-deprecating language, excessive self-criticism, such as making negative comments about oneself or showing constant feelings of guilt [7,15,17].

In interpersonal relationships, these individuals, according to people who know them best, also show difficulty in maintaining eye contact and initiating or sustaining conversation. Interestingly, not all are described as quiet or reserved; on the contrary, some are described as talkative, outgoing, and confident. In some cases, however, this talkativeness is described as excessive and socially inappropriate, a sign of nervousness or difficulty interpreting social contexts [15].

Regarding the life experiences that seem to unite individuals complaining of lonely conditions, the literature points to heterogeneous past and/or present adversities and challenging events, such as lack of work or difficulty in finding it; economic difficulties; conflicting family relationships or family members with serious mental or physical health problems; frequent moves, often to other countries, resulting in loss or difficulty maintaining relationships and meeting with friends and relatives; frequent school or job changes resulting in a sense of instability and uncertainty and often accompanied by the pressure to adapt quickly to a new and unfamiliar environment without their own points of reference, such as some former teachers or former colleagues, who helped them cope with their relational difficulties [7,15,50].

Another important aspect highlighted by the literature is the reluctance of many people who complain of loneliness to seek help either from family members, who are often perceived as more distant than they actually are, or from professionals such as psychologists, psychotherapists, etc. to overcome this unpleasant experience. The reasons seem to be many, from a lack of trust in others and/or the habit of considering this condition as part of one’s nature and, therefore, unchangeable, to the belief that one is hopeless and that any attempt to improve the situation will be a failure [15].

In other cases, some people who ask for and receive some form of help do not appear fully involved and are reluctant to act and, in fact, benefit from such support [15,41]. This further demonstrates the complexity of the phenomenon under consideration and the difficulty of successfully addressing it without a thorough investigation of the potential protective and risk factors that act in giving it certain forms and characteristics.

## 5. Conclusions

In conclusion, the results of our investigation seem to confirm the initial hypotheses and the assumptions in the relevant literature, underscoring the central role that PTSD and DSO symptoms, as well as maladaptive personality traits, may have on adolescents’ perceptions of loneliness, affecting their psychological well-being.

### 5.1. Limitations

The research is subject to a number of limitations, primarily the cross-sectional nature of the design, which precludes the possibility of drawing causal inferences among the variables under examination. Indeed, cross-sectional models, by their inherent characteristics, can only represent a snapshot of a specific moment in time in the much broader path and operation of the variables investigated. To confirm the validity of the selected mediating model, future research should use longitudinal or experimental designs, which would provide a more complete and detailed understanding of the interaction between the variables examined. Such models, in fact, allow for better evaluation of the results derived from the cross-sectional model, enabling causal inferences to be drawn and providing stronger evidence for the existence and functioning of the hypothesized relationships between the variables analyzed. A further significant limitation is the exclusive use of self-report instruments, without qualitative or confirmatory diagnostic interviews. Although self-report instruments are undoubtedly the easiest and fastest method of data collection, they are not without inherent limitations. Self-reported responses may be exaggerated, respondents may be too embarrassed to reveal personal details, and various biases may influence the results. These include social desirability and social appreciation bias, indulgence bias, acquiescence bias, and the need for consistency and rationality [64]. In addition, the failure to understand some words, the presence of sentences expressed in a negative form, structured in a particular way, or phrased in language that could give rise to ambiguous or ambivalent interpretations, could lead to difficulties in responding by participants who might read questions quickly or with distractions, or be influenced by the framing effect [64]. The role of personal memories and emotions in the interpretation of events, particularly those from the past, can also be a source of frequent and sometimes significant bias in participants’ approach to questionnaires. It would be appropriate to improve these instruments through the inclusion of qualitative methods and/or clinical interviews with the aim of reducing the typical limitations of self-report instruments. Again, a useful strategy for reducing this type of bias is the implementation of a longitudinal design to mitigate the inherent limitations associated with self-report interviews and to gain a greater understanding of the nature and directions of the relationships between the variables being investigated.

In addition, it would be advantageous to expand the study population through the involvement of a sample of individuals older than 17 years, particularly emerging and young adults, in order to obtain a more complete understanding of the phenomenon under analysis and to monitor the characteristics and dynamics it may acquire in the transition between adolescence and adulthood. Observation of a population of other age groups would allow us to observe the evolution of the phenomenon or its possible later relapse episodes in relation to maladaptive personality traits, which are more stable and defined in adulthood, and to delve into the diachronic dimension of the role of PTSD and/or DSO symptomatology related to traumatic and adverse events experienced later in life or, if they occurred in childhood and adolescence, to verify their effects at a later date.

### 5.2. Future Directions

The results of the present study, despite the important limitations mentioned above, may provide insights for the design and testing of clinical, social, or educational interventions to address the perception and condition of loneliness among adolescents.

Loneliness is increasingly recognized as a major health and social problem, so much so that some health professionals have called it an epidemic. Initiatives have been launched around the world to address the “loneliness epidemic”, and in 2018, the United Kingdom named the world’s first minister for loneliness [41].

As loneliness is increasingly considered a public health problem, scholars say public health approaches are needed to address it, starting with defining the extent and distribution of the problem in different settings, environments, and age groups. With this in mind, it may be important to explore the potential risk and protective factors related to loneliness in order to make interventions to prevent and manage it more effectively and timelier, and to identify strategies more targeted to the specific characteristics of individuals experiencing chronic and pervasive loneliness [5,7,16]. Schools, for example, should monitor children who are friendless or lonely and pay attention, with regard to adolescents, to potential dysfunctional behaviors that might emerge as a way to cope with feelings of loneliness [7,27].

Our research design is inspired by the hypothesis, derived from the reviewed literature, that a better understanding of potential factors that may be involved in the exacerbation of experiences of loneliness may be useful for the development and promotion of more targeted prevention interventions. Because our study highlights, among the factors that undermine an individual’s ability to interact and integrate with the social context, family environment, peers, etc., the significant role of various maladaptive personality traits and dysfunctional coping strategies in relation to the adversity and trauma experienced, it is recommended that researchers and clinicians dealing with pervasive and chronic loneliness conditions consider the importance of personality traits and early recognition of the presence of PTSD- and DSO-related symptoms which appear to have a significant impact on perceptions of loneliness, in order to prevent this condition, which is somehow intrinsically linked to adolescence, from taking on dysfunctional characteristics and transforming over time into severe distress, isolation, and social avoidance that can seriously impair adolescents’ psychological and social development.

On the basis of our findings, we believe that paying attention to adolescents’ communication and interpersonal skills and their ability to respond functionally to stressful and traumatic events, designing and implementing interventions aimed at improving the social and interpersonal skills of those who are at risk or are facing this unpleasant experience, may facilitate the identification of more targeted strategies that, as such, may be more successful in preventing and overcoming the perception of loneliness and related discomforts, which are often risk or maintenance factors [4,20,25].

The results of this study can be generalized to adolescents who report feelings of loneliness and have symptoms of post-traumatic stress disorder and/or self-organization disorders accompanied by maladaptive personality traits.

## Figures and Tables

**Table 1:** Descriptive Analyses, Reliability, and Correlations.

	M	SD	*S*	*K*	*α*	1	2	3
1. Maladaptive Personality	25.8	11.8	0.37	0.30	0.87	-	-	-
2. Loneliness perceived	45.2	10.6	0.19	−0.24	0.90	0.55 *	-	-
3. Post-Traumatic Stress disorder	15.9	8.4	0.005	−0.56	0.86	0.57 *	0.47 *	-
4. Disturbance of self-organization	13.6	8.3	0.41	−0.54	0.87	0.63 *	0.67 *	0.70 *

Note: *N* = 901. * *p* < 0.01.

**Table 2:** Goodness-of-fit indices of the measurement models.

	χ^2^	*p*	CFI	TFI	SRMR	RMSEA
Fit Indices	(48) = 323.30	<0.01	0.97	0.95	0.03	0.08

**Table 3:** Path Estimates of the structural model, SE, and 95% CIs. The following table shows the values of standardized beta coefficients, standardized errors, *p*-values, and maximum and minimum levels of confidence intervals.

**Direct Effect**	***β***	***p***	**SE**	**CI LL**	**CI UL**
MP → PTSD	0.20	<0.001	0.02	0.04	0.14
MP → DSO	0.55	<0.001	0.03	0.19	0.29
LP → MP	0.25	<0.001	0.07	0.21	0.48
LP → PTSD	−0.20	<0.001	0.03	−0.19	−0.06
LP → DSO	0.74	<0.001	0.04	0.35	0.53
**Indirect Effect of PTSD via MP**	***β***	***p***	**SE**	**CI LL**	**CI UL**
PTSD → LP	0.05	<0.001	0.01	0.01	0.05
**Indirect Effect of DSO via MP**	***β***	***p***	**SE**	**CI LL**	**CI UL**
DSO → LP	0.14	<0.001	0.05	0.05	0.12

Note: MP = Maladaptive Personality traits; PTSD = Post-traumatic stress disorder; DSO = Disturbance of self-organization; LP = Loneliness Perception; β = standardized beta coefficient; *p* = level of significance; SE = standard error BC 95%; CI = confidence interval; LL = lower limit; UL = upper limit.

## Data Availability

The raw data supporting the conclusions of this article will be made available by the authors upon request.
